# Association Between a Woman's Age at First Birth and High Blood Pressure

**DOI:** 10.1097/MD.0000000000000697

**Published:** 2015-04-24

**Authors:** Joanne M. Lind, Annemarie Hennessy, Christine L. Chiu

**Affiliations:** From the University of Western Sydney, School of Medicine, Sydney, New South Wales, Australia.

## Abstract

The aim of this article is to determine whether the age of a woman at first birth is associated with treatment for high blood pressure (HBP) later in life.

Baseline data for 62,914 women were sourced from the “45 and Up Study,” an observational cohort study of healthy aging in Australia. These women had given first birth between the ages of 18 and 45 years. Odds ratios and 95% confidence intervals for the association between age that a woman gave first birth and treatment for HBP were estimated using logistic regression. Data were stratified by current age (<60 and ≥60 years) and adjusted for demographic and lifestyle factors.

There was a significant association between age at first birth and present day HBP. Older age at first birth was associated with a lower likelihood of HBP in women aged 25 to <35 years and 35 to 45 years at first birth (in women currently <60 years) and 35 to 45 years at first birth (in women currently ≥60 years of age), compared with women aged 18 to <25 years at first birth, adjusting for demographic and lifestyle factors.

Women who were older when they gave first birth had lower odds of treatment for HBP compared with women who were younger when they gave birth to their first child. The contribution of a woman's pregnancy history, including her age at first birth, should be discussed with a patient when assessing her risk of HBP.

## INTRODUCTION

Cardiovascular diseases, including hypertension, are the leading cause of death in developed countries. High blood pressure (HBP) is a major contributor to an individual's risk of developing other cardiovascular diseases including atherosclerosis, myocardial infarction, and stroke. Risk factors for hypertension include a positive family history, high body mass index (BMI), low sociodemographic level, and low levels of physical activity. A gender difference is evident in blood pressure recordings, with women having lower systolic blood pressures compared with men, during early adulthood, but the prevalence of hypertension in females exceeds that of men in older adults.^1^ Recent studies have shown that female-specific activities, including lactation,^2^ HBP during pregnancy,^3^ and use of menopausal hormone therapy (MHT),^4,5^ are all associated with altering a woman's likelihood of having HBP in later life.

A number of physiological changes occur during normal pregnancy that place increased stress on the cardiovascular system, with the uterus and placenta consuming >20% of a woman's cardiac output.^6^ Despite these changes, a woman's arterial pressure decreases during midpregnancy,^7,8^ which is accompanied by a dilation of her microvasculature. The long-term consequences of these acute changes to a woman's cardiovascular system are relatively unknown. Studies have shown that the age a woman is when she gives first birth is associated with longevity, with the age at first birth higher in women who live longer.^9^ Many other studies, over many decades, have shown an association between the age a woman is when she first gives birth and her subsequent risk of cancer, with younger primiparous women having a decreased risk of future cancer.^10–13^ No studies have investigated whether a woman's age at first birth alters her likelihood of future HBP.

The present study aimed to investigate the relationship between the age at first birth and prevalence of HBP using observational data from the 45 and Up Study, Australia.

## MATERIALS AND METHODS

Data was obtained from women who were recruited from the 45 and Up Study. The methods for the study have previously been described elsewhere.^14^ Briefly, the 45 and Up Study is a large-scale cohort study of healthy aging that involves 267,153 men and women aged ≥45 years from the general population of New South Wales, Australia. Participants were randomly selected from the Australian National Universal Health Insurance Database (Medicare). The participants were enrolled into the study by consenting to and completing a baseline questionnaire (available at www.saxinstitute.org.au/our-work/45-up-study/) and giving consent for long-term follow-up through data linkage and repeat data collection. People aged ≥80 years and residents of rural and remote areas were oversampled. Study recruitment commenced in January 2006 and was completed in April 2009. Ethical approval for the 45 and Up Study was obtained from the University of NSW Human Ethics Committee and the current study was also approved by the University of Western Sydney Human Research Ethics Committee.

**FIGURE 1:**
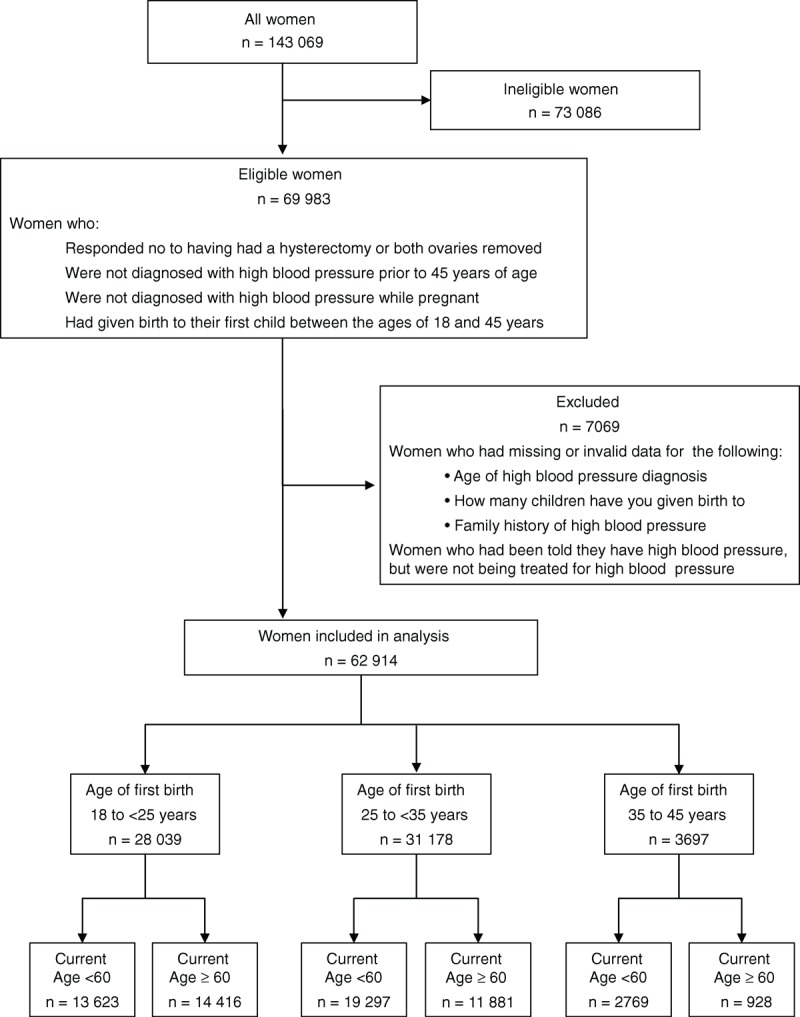
Participants included in the study.

All of the data used in this study were acquired from the 45 and Up Study baseline questionnaire. Women were included in this study if they were of age ≥45 years, had given birth between 18 and 45 years of age, had not had a hysterectomy or both ovaries removed, were not diagnosed with HBP prior to the age of 45 years, and were not diagnosed with HBP during pregnancy (Figure 1).

Women were defined as being treated for HBP if they answered “Yes” to the question “In the last month have you been treated for: HBP.” Women were excluded if they answered “Yes” to “Has a doctor ever told you that you have: HBP—when not pregnant,” but were not being treated for HBP; they failed to provide an age of onset for HBP; they provided invalid data for family history; or they provided invalid data for the number of children they had given birth to in their specified age range (Figure 1).

Classification of demographic and lifestyle characteristics have been described elsewhere.^5^ Briefly, for “Country of Birth,” participants were classified according to whether they had been born in Australia or overseas. Physical activity levels were assessed using questions from the Active Australia Survey, including the number (and duration) of moderate, vigorous, and walking activities. Weighted weekly average physical activity duration was calculated, with vigorous activity receiving twice the weighting of moderate or walking activity, and was categorized into sufficient (>150 min/wk) or insufficient levels.^15,16^ Participant data was stratified into 2 age categories (<60 and ≥60 years) using the median age of the cohort as the cut point. Age at first birth was stratified into 3 categories (18 to <25; 25 to <35; and 35 to 45 years).

### Statistics

Ordinal logistic regression was used to determine the association between age at first birth and sociodemographic variables. Binary logistic regression was used to generate odds ratios (ORs) and 95% confidence intervals (CIs) for the association between age at first birth and HBP in later life. Both crude and adjusted ORs were calculated and descriptions refer to adjusted ORs unless otherwise specified. Owing to the known association between HBP and aging, women were stratified according to age using the median age as the cut point (<60 and ≥60 years) when testing the association between age at first birth and HBP in later life. ORs were adjusted for current age (as a continuous variable), country of origin, income level, BMI, smoking status, alcohol consumption, physical activity, family history of HBP, history of MHT, number of children, and whether a woman breastfed. Categories for each covariate have been previously described.^5^ An additional category for missing values was included for variables with missing data. All statistical tests were 2-sided, using a significance level of *P* < 0.05. All statistical analyses were carried out using SPSS software version 20 (IBM Corp, Armonk, NY).

## RESULTS

A total of 62,914 women were included in the analysis. There was an association between present day demographic and lifestyle factors and the age a woman gave first birth. Women who were of an older age when they gave first birth were more likely to be currently <60 years of age; have been born outside Australia; have a higher present day income; have a lower BMI; have never smoked; consume more alcohol per week; be less physically active; have never used MHT; have had less children; and to have breastfed (adjusting for all other demographic and lifestyle factors in the model). There was no association between having a family history of HBP and the age a woman gave first birth (Table 1).

**TABLE 1:**
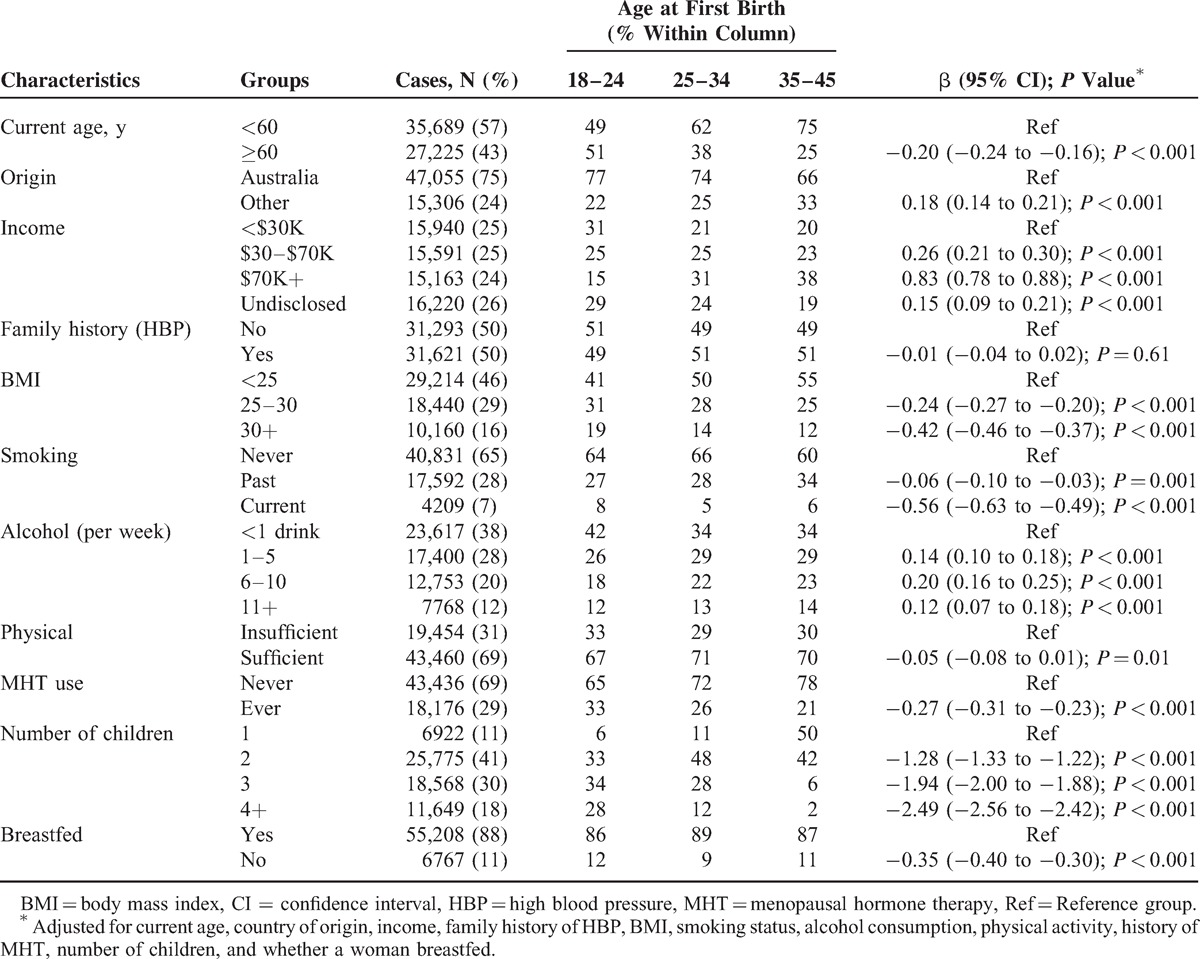
Sociodemographic Factors Associated With Age at First Birth

The prevalence of treatment for HBP decreased as a woman's age at first birth increased with 16.0% of women reporting current HBP if they gave birth between the ages of 18 and <25 years; 11.9% reported HBP if they gave birth between the ages of 25 and <35 years; and 7.5% of women reported HBP if they gave birth between the ages of 35 and 45 years.

**FIGURE 2:**
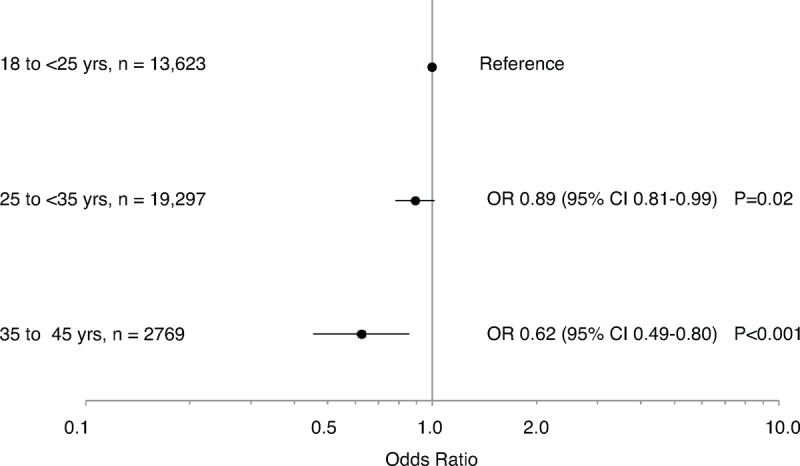
Age at first birth and high blood pressure in women aged 45 to <60 years. Data are presented as odds ratios (ORs) and 95% confidence intervals (CIs), adjusted for current age, country of origin, income level, body mass index, smoking status, alcohol consumption, physical activity, family history of high blood pressure, history of menopausal hormone therapy, number of children, and whether a woman breastfed.

**FIGURE 3:**
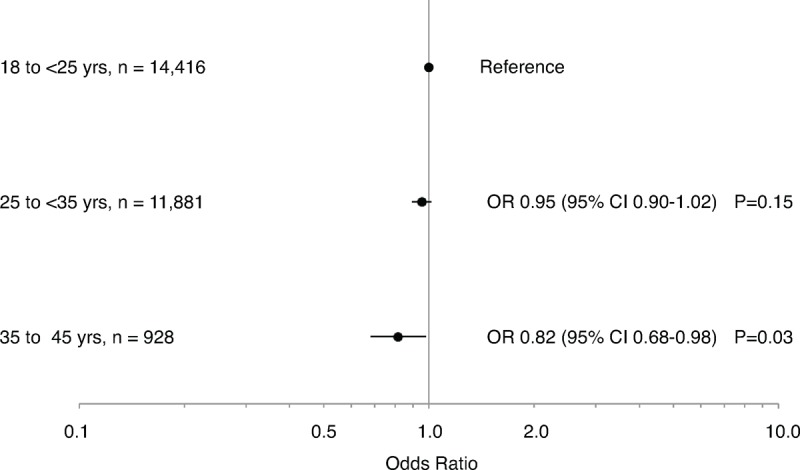
Age at first birth and high blood pressure in women aged ≥60 years. Data are presented as odds ratios (ORs) and 95% confidence intervals (CIs), adjusted for current age, country of origin, income level, body mass index, smoking status, alcohol consumption, physical activity, family history of high blood pressure, history of menopausal hormone therapy, number of children, and whether a woman breastfed.

Once stratified by current age, there was a significant association between age at first birth and present day HBP in women <60 years of age (25 to <35 category: crude OR 0.68, 95% CI 0.62 to 0.74, *P* < 0.001; 35 to 45 category: crude OR 0.41, 95% CI 0.33 to 0.52, *P* < 0.001; compared with women aged 18 to 25 years at first birth). There was also a significant association between age at first birth and present day HBP in women ≥60 years of age (25 to <35 category: crude OR 0.94, 95% CI 0.88 to 0.99, *P* = 0.02; 35 to 45 category: crude OR 0.79, 95% CI 0.67 to 0.93, *P* = 0.005; compared with women aged 18 to 25 years at first birth). After adjusting for lifestyle and demographic factors, a significant association remained in women <60 years of age in the 25 to <35 category and 35 to 45 category (Figure 2), and in women ≥60 years of age in the 35 to 45 year category (Figure 3), with increased age at first birth associated with a decreased likelihood of present day HBP.

## DISCUSSION

This study aimed to investigate the association between a woman's age at first birth and her likelihood of having HBP in later life. Women who gave birth to their first child between 25 and 45 years had a lower likelihood of present day treatment for HBP compared with women who gave birth to their first child between the ages of 18 and <25 years. This association remained after stratifying by current age and adjusting for demographic and lifestyle factors.

These analyses were carried out in Australia's largest study of aging individuals, the 45 and Up Study. A limitation of the study is that the data used was self-reported and may be prone to recall bias. The causal nature of the association cannot be determined as the data were cross-sectional. One possible explanation for the findings is that women who were older when giving birth to their first child were more likely to be healthier and in a high socioeconomic group, demonstrated by significantly higher present day incomes, significantly lower levels of obesity, and a significantly lower prevalence of smoking (Table 1). These women were, however, less likely to have sufficient levels of physical activity and were more likely to consume >1 alcoholic drink per week. After adjusting for all variables outlined in Table 1 in the statistical modeling, the association remained significant for both women aged <60 years of age and women ≥60 years of age.

This is the first study to show an association between the age at first birth and the likelihood of treatment for HBP in later life. A number of studies have examined the effects of pregnancy on longer term cardiac status and it has been postulated that pregnancy leads to a favorable effect on future potential cardiovascular disease.^17^ There are several large-scale population studies that show there is a disadvantage in a higher number of pregnancies and increased interpregnancy interval on long-term hypertension and cardiovascular risk.^18^ This includes data in Aboriginal communities in Australia^18^ showing that >3 pregnancies predisposes to hypertension and heart disease, a finding also seen in the large-scale Swedish population linkage studies.^19^ Pregnancy favorably not only affects systemic vascular resistance and cardiac output but also induces changes in lipid profiles that may be atherogeneic.^20^ The cardiovascular adaptation to pregnancy is a profound one with possible long-term consequences. This study shows that the older a woman is when she gives first birth (up to 45 years of age) is associated with a reduced likelihood of future HBP. Detrimental effects of pregnancy on cardiovascular health may be more evident in women who first gave birth at a younger age (18 to 25 years), as they spend a greater proportion of their life as a parous woman, and therefore there is a longer period of time for any long-lasting effects of pregnancy to become evident. Previous studies have linked increased longevity to an older age at first birth.^9^ Increased longevity may, in part, be attributed to a lower likelihood of HBP in this same set of women.

These results provide further evidence that a woman's pregnancy history is important when calculating her likelihood of future cardiovascular disease, specifically HBP. The contribution of a woman's pregnancy history, including her age at first birth, should be discussed with a patient when assessing hypertension risk.

## Acknowledgements

*This research was completed using data collected through the 45 and Up Study* (*www.saxinstitute.org.au*).*The 45 and Up Study is managed by the Sax Institute in collaboration with major partner Cancer Council NSW; and partners: the National Heart Foundation of Australia (NSW Division); NSW Ministry of Health; beyondblue; Ageing, Disability and Home Care, Department of Family and Community Services; the Australian Red Cross Blood Service; and UnitingCare Ageing. The authors thank the many thousands of people participating in the 45 and Up Study*.
